# Staff Successes and Challenges with Telecommunications-Facilitated Patient Care in Hybrid Hospital-at-Home during the COVID-19 Pandemic

**DOI:** 10.3390/healthcare11091223

**Published:** 2023-04-25

**Authors:** Stephanie K. Zawada, Jeffrey Sweat, Margaret R. Paulson, Michael J. Maniaci

**Affiliations:** 1 Mayo Clinic Graduate School of Biomedical Sciences, Mayo Clinic College of Medicine and Science, 13400 E. Shea Blvd., Scottsdale, AZ 85259, USA; 2 Social Science Department, University of Wisconsin-Stout, 712 Broadway St. S, Menomonie, WI 54751, USA; 3 Mayo Clinic Health System, 1221 Whipple St., Eau Claire, WI 54701, USA; 4 Mayo Clinic Florida, 4500 San Pablo Rd., Jacksonville, FL 32224, USA

**Keywords:** home hospital, hybrid care, telehealth, mobile integrated technology, staff satisfaction, rural healthcare, paramedic, ancillary health professionals, allied health

## Abstract

Technology-enhanced hospital-at-home (H@H), commonly referred to as hybrid H@H, became more widely adopted during the COVID-19 pandemic. We conducted focus group interviews with Mayo Clinic staff members (n = 14) delivering hybrid H@H in three separate locations—a rural community health system (Northwest Wisconsin), the nation’s largest city by area (Jacksonville, FL), and a desert metropolitan area (Scottsdale, AZ)—to understand staff experiences with implementing a new care delivery model and using new technology to monitor patients at home during the pandemic. Using a grounded theory lens, transcripts were analyzed to identify themes. Staff reported that hybrid H@H is a complex care coordination and communication initiative, that hybrid H@H faces site-specific challenges modulated by population density and state policies, and that many patients are receiving uniquely high-quality care through hybrid H@H, partly enabled by advances in technology. Participant responses amplify the need for additional qualitative research with hybrid H@H staff to identify areas for improvement in the deployment of new models of care enabled by modern technology.

## 1. Introduction

Patient care at home has a long history, and it was not until the 20th century that “house calls” were abandoned in favor of patient care in hospitals across the industrialized world, driven by changes in technology, financial considerations, and liability [1]. While brick-and-mortar hospital care comes with many advantages, concerns regarding cost, overcrowding, patient satisfaction, and hospital-associated infections existed long before the COVID-19 pandemic [1,2]. The role of interprofessional health staff in preventing complications and improving the safety of admitted patients, as well as their satisfaction and long-term health outcomes, has been the subject of an increasingly significant area of health services research [2]. One healthcare delivery model uniquely successful at reducing hospital-associated complications for chronic disease patients requiring inpatient-level care for an acute episode is the hospital-at-home (H@H) program, early versions of which were launched in the United States over 20 years ago [3]. Since its debut, the H@H model has not disappointed, with multiple studies finding that the program is associated with better patient outcomes and satisfaction compared to brick-and-mortar care [4,5,6]. Widespread adoption of the program, however, was never realized in the US, in part due to limited payer reimbursement [7]. During the COVID-19 Public Health Emergency (PHE), the Centers for Medicare and Medicaid Services (CMS) introduced a waiver, the Acute Hospital Care at Home (AHCaH) waiver, permitting hospitals participating in Medicare to temporarily forgo the federal standard for Conditions of Participation (CoPs) that stipulates 24/7 on-site nursing staff be available for inpatient care [8]. This waiver allowed eligible patients to receive hospital-level care at home and providers to receive reimbursement for home hospital services identical to those delivered in a brick-and-mortar setting, provided that 24/7 clinical monitoring was performed using remote monitoring and telehealth [9]. Thus, regulatory reforms implemented during the COVID-19 pandemic generated momentum to deploy H@H programs across the US [10].

Traditional hospital-at-home (H@H) programs that deliver acute, or inpatient-level, hospital care reduce the likelihood of adverse clinical events, such as bacterial infections, and overcome limited hospital bed constraints. The acute care interventions delivered via H@H programs include in-home paramedic visits, in-home nurse visits, mobile imaging, point-of-care testing, and intravenous medications. Some programs also include the delivery of medical supplies and prescriptions. Over the past few years, a modern model for H@H has developed, commonly referred to as the hybrid H@H model. Hybrid H@H programs combine the traditional in-home healthcare visits with 24/7 access to hospital staff via mobile tablet or smartphone-enabled telehealth, including but not limited to video telehealth, remote monitoring, and electronic messaging communications.

While the majority of research published on H@H programs focuses on “low tech” care, a growing body of literature evaluating hybrid H@H for acute care has emerged [11]. To date, only one randomized clinical trial for hybrid H@H with 172 acute patients, conducted before the COVID-19 pandemic, has been published. Comparing in-person to telehealth visits during participation in the program, researchers found that telehealth visits were not inferior to in-person visits at home [12]. In a more recent qualitative analysis of a small, randomized control trial (n = 36) conducted before 2020, researchers reported that some patients in the hybrid H@H program “had better experiences with their care team, had more experiences promoting healing, such as better sleep and physical activity, and had better experiences with systems factors, such as the admissions process” [13]. Another qualitative study assessed hybrid H@H implementation during the COVID-19 pandemic, identifying implementation barriers through semi-structured interviews with program leaders [14]. The current dearth of data from robust randomized clinical trials for evaluating the effectiveness of hybrid H@H compared to both traditional H@H and in-hospital care remains a barrier to securing permanent reimbursement models necessary to advance research in this area. Furthermore, hybrid H@H programs vary across hospitals, with the list of telehealth services available dictated by state regulations. Currently, no research has been published regarding the experiences of frontline staff in deploying a single hybrid H@H model in rural, urban, and desert settings. Addressing this lack of evidence is critical to ensure the 2024 assessment report on the AHCaH waiver by the US Department of Health and Human Services is robust and considers the frontline implementation experiences of staff. 

To understand the experiences of hybrid H@H staff in diverse environments during the COVID-19 pandemic, we conducted a focus group interview study of staff members in the Mayo Clinic hybrid H@H program, which consists of comparable services delivered in other studied hybrid H@H programs, including at-home twice-daily nurse visits, intravenous medications, remote monitoring, video telehealth, and point-of-care testing, with the distinct structural difference being that, while most hybrid H@H programs are limited geographically to a single state and its licensed providers, Mayo Clinic’s command center for H@H across states is located at Mayo Clinic Florida. We assess our findings in the context of broader healthcare staffing issues as it relates to experiences in implementing a new care delivery model and experiences with new technology to monitor patients at home to outline new areas for qualitative research in hybrid H@H programs and consideration by policymakers and payers. 

## 2. Materials and Methods

### 2.1. Mayo Clinic’s Hybrid H@H Model

**Figure 1:**
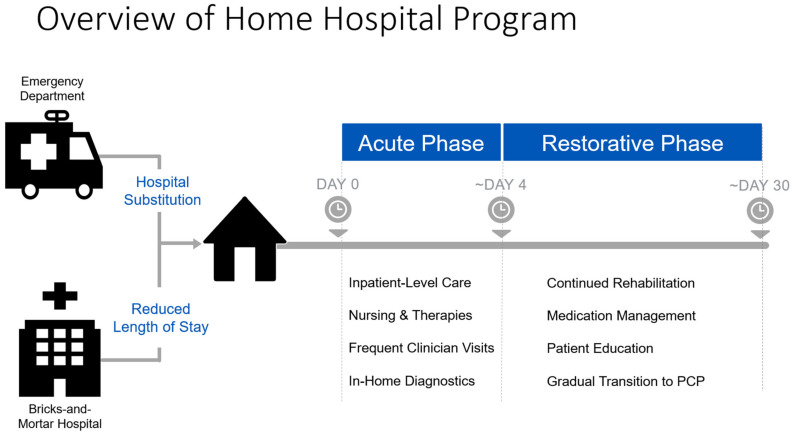
Overview of Mayo Clinic ACH model.

In the summer of 2020, Mayo Clinic launched its hybrid H@H program: Advanced Care at Home (ACH) (Figure 1). ACH was implemented via a collaboration with a Boston-based partner, Medically Home, who specializes in the delivery of inpatient level at home using cutting-edge technology, under the AHCaH waiver. ACH was first launched at Mayo Clinic Health System, a rural community practice in northwest Wisconsin (NWWI), then at an urban destination medical center located in Jacksonville, Florida, the Mayo Clinic Florida (MCF) campus, and finally, at the North Phoenix/Scottsdale Mayo Clinic Hospital (MCA). The command center for the tri-site ACH program, staffed with 24/7 clinical personnel, is located at MCF. Eligibility criteria for patients to receive acute care at home under the waiver required that patients be assessed in person, at a hospital, to obtain history and physical examinations. Conditions eligible for management after an acute episode via this program include, but are not limited to, a wide range of conditions, such as urinary tract infection, influenza, and asthma exacerbations [15]. Social and domestic screening was also required to assess whether a patient could successfully participate in hybrid H@H without undue hardship, such as residing in a location less than 30 min away from nursing and paramedic staff. Patients comfortable with the use of mobile tablets and smartphones for telehealth and virtual messaging who met all eligibility criteria and screening measures were approached by clinical staff and offered enrollment in the program.

While site license limitations at MCF and MCA required the use of an outsourced supplier model to facilitate at-home care, the NWWI community practice used an insourced supplier network to provide home health, nursing, pharmacy, and paramedicine services. Patients in ACH received a proprietary technology package, with the following wireless-enabled tools to record and transmit frequent biometric data for remote monitoring: a blood pressure cuff, a pulse oximeter, a thermometer, and a scale. Patients also received a Bluetooth-enabled mobile tablet for 24/7 access to the MCF Command Center via video or text-based chat.

### 2.2. Study Recruitment

All ACH staff (45), excluding study investigators (M.R.P. and M.J.M.), were invited to participate in the focus group interviews. Research coordinators for the ACH program at MCF, MCA, and NWWI sent multiple interview invitations from July 2021 to February 2023. Research coordinators worked with staff to find interview blocks where staff members could meet via video teleconference with qualitative researchers to facilitate focus groups. This study was approved by the Mayo Clinic Institutional Review Board under protocol number 20-010753.

### 2.3. Focus Group Interviews

In selecting an interview method, the small size of the ACH program, with an average of 45 staff members running the inpatient-level program 24/7, was a critical limitation for scheduling interviews. Given the highly collaborative nature of the program and its reliance on virtual communication, including videoconferencing, the focus group interview method was used to tap into the day-to-day interaction among staff and to encourage staff participation from those who might be reluctant to be interviewed on their own or who might feel they have little to say [16]. 

Limited by the total number of staff on the floor needed to deliver care, the maximum session time was set at 90 min to complete the interview, although interview length could be extended by the interviewers to ensure that all questions could be answered thoroughly by the staff.

Participating staff were informed that the interviews were confidential, and that their responses would be deidentified. The interviews conducted for this study included open-ended question prompts (Appendix A), allowing staff to raise issues of importance and use their own terminology to discuss these issues with colleagues [16].

Six interviews were conducted by qualitative researchers unaffiliated with the ACH program (S.K.Z. and J.S.). To facilitate staff discussion, interviewers asked questions to each participating staff member individually, followed by restating the question to the group at large to obtain consensus. If participating staff joined late or left early, the question was only presented to staff in attendance. When participants elected to share an experience or theme independent of question prompts, an interviewer asked probing questions to obtain additional relevant information. Interviews were conducted virtually using the Zoom or Microsoft Teams software platform. 

### 2.4. Data Analysis

Interviews were transcribed by Mayo Clinic external partners. Two study investigators (S.K.Z. and J.S.) qualitatively analyzed interview transcripts, guided by grounded theory’s iterative methods [17]. With grounded theory, investigators used an inductive approach to develop theories on the basis of evidence from data collection and analysis. Given the unprecedented situation in which the hybrid H@H program was deployed, namely, the COVID-19 pandemic, the use of deductive methods to analyze interview data was unsuitable. In contrast, a grounded theory approach was appropriate to study novel experiences unique to this program during the pandemic. 

After n = 3 transcripts (21%), which were representative of our sample and included staff from multiple sites, were coded to consensus using an open coding process by two investigators (S.K.Z. and J.S.), one investigator (S.K.Z.) used an axial coding process to generate a codebook of inductively identified categories (Appendix B). After a review of the codebook by two study investigators (M.J.M. and M.P.), NVivo 12 was used to individually code each transcript (n = 14). All investigators were involved in the review of coding and analysis to ensure accuracy and consistency across study sites for the program. Through coding, themes were identified from participant stories and examined through the lens of the study’s goals: to explore staff experiences in implementing a new hybrid care delivery model and in using new technologies to deliver care. Quoted statements were edited for readability when necessary. Here, we present themes related to the experiences of hybrid H@H staff at rural, urban, and desert medical centers during the COVID-19 pandemic.

## 3. Results

### 3.1. Demographics

Six separate focus group interviews were conducted between August 2021 and February 2023. By the end of the study, 14 staff members had been interviewed (Table 1). Among participants, seven (50%) were from MCF, five (35.7%) were from NWWI, and two (14.3%) were from MCA. To protect the confidentiality of staff participating in a small pilot program within a global institution, limited participant demographic information was obtained, and participants self-reported their staff role. Interview length ranged between 30 and 70 min, with the average interview length of 45 min.

### 3.2. Experiences in Implementing a New Telecommunications-Facilitate Care Delivery Model

Theme 1: Quality of Care

A main concern about H@H programs is whether the care provided is equivalent in quality to care received in a brick-and-mortar hospital. A known benefit of recuperating at home is reduced sleep disruption [18]. Furthermore, telehealth, a core component of hybrid H@H programs such as ACH, has enabled providers to tailor healthcare experiences to meet the needs of individual families [19]. In our interviews, ACH staff unanimously expressed that many patients in ACH were receiving care on par with or exceeding the quality of in-hospital care.

When I think about the successes that we’ve had, we’ve served hundreds of patients now in a virtual hospital with a high severity of illness. These aren’t observation-level patients—they’re pretty sick. We have had generally excellent patient experience and stellar clinical outcomes. Our clinical outcomes in almost all arenas are far better than the same cohort of patients in the hospital. There’s some more research that needs to be performed in a randomized controlled trial setting, but, when we look at some of the initial information, it is a successful program.

*—Nurse Manager, Leadership Staff Member, Wisconsin*

Improved clinical outcomes in in-hospital care are associated with low patient-to-nurse ratios, which lead to lower mortality and failure-to-rescue rates compared to hospitals with less nursing staff [20]. The ACH model’s remote monitoring-heavy approach ensured that multiple layers of staff participated in patient oversight. When expanding on why, in some situations, staff could deliver higher-quality care in ACH than in the hospital, some staff members noted that more accessible leadership and colleagues played a role:

There’s a lot of leadership presence, I would say, across all the sites, so I think we probably catch things faster and quicker in ACH than in the brick and mortar. Having run a brick-and-mortar inpatient unit, and having worked in one, I would imagine that more things are just discovered, I think, in ACH.

*—Nurse Manager, Leadership Staff Member, Florida*

In brick and mortar, you’re able to handle things on your own. You don’t need to go through one to two or three other people to get something done for your patient. Generally, you just ask your provider. They OK, and boom, it’s done. But this is a little bit different. You have to break that way of thinking, be able to plan out your day, delegate to the right person, work as a team. It’s a very, very big team environment, here in the department, which is very, very good because we’re all working in the command center, whereas working on the floor you have an idea that you’re alone and you don’t want to bother anyone. [Working in the command center], you can see how your coworker’s doing. If they’re having a little bit of a rough day you can ask, “Hey, can I take anything off your plate?”, and that is a general cultural here which is really awesome.

*—Nurse Coordinator, Frontline Staff Member, Florida*

By facilitating the observation of in situ experiences of patients receiving inpatient-level care at home, ACH is improving outcomes for patients in the hospital, as well as enhancing the practice of medicine at Mayo Clinic.

It’s across the true continuum of care. You’re seeing the patient whether they come in straight from the ER or we’re taking them from the inpatient setting. They’re stabilized, but still ill enough to come home because you still have to meet inpatient criteria. What does their life look like at home? How do we have to adapt? We’ve learned a lot about patients and what they do when they go home, and that we are making these patients’ regimens upon discharge too challenging for the average elderly, male or female, to follow. And so, we need to work on that. They’re taking too many medications at too many complicated times. And then we wonder why our heart failure patient comes back to the hospital because I don’t know that I could follow some of these complex regimens. I think that’s what we’re learning a lot about, medicine, and what we’re doing in the home hospital is actually impacting what we do in the brick and mortar.

*—Nurse Administrator, Leadership Staff Member, Florida*

The amount of extra information you get from being in their home that you don’t get when they’re in the hospital is just profound. Seeing that bag of salty potato chips sitting on their counter saying, “Well, that’s probably one of the reasons why you have a heart failure exacerbation: due to noncompliance with diet.” That’s something that I wouldn’t get if I was not in their home, and so things like that are just so much more valuable. And you can get that really personalized aspect of care through this program that you can’t get in the brick-and-mortar hospital.

*—Physician, Leadership Staff Member, Arizona*

Multiple staff members stated that patient satisfaction in ACH was high and provider satisfaction was unique. For example, one staff member reflected on the role of ACH during the holiday season in 2020:

With COVID-19 and everything else going on, we had 10-plus patients that would have been in an isolation room by themselves on Christmas, and they were able to be home with their families. And so, that’s a feel good, right? You have to think about those things.

*—Nurse Manager, Leadership Staff Member, Wisconsin*

One factor associated with higher-quality care delivered by ACH, and the associated unique patient satisfaction experience, is the finding that ACH expanded patient choice. Zolkefli (2017) found that offering patients choices is “essential to good clinical care because the patient’s cooperation and satisfaction reflect the degree to which medical intervention fulfills his or her choices, values, and needs” [21]. By allowing patients to receive care at home, ACH put the personal wants and needs of patients first.

I think [ACH is] giving patient choice back, because traditionally, you’re admitted; you don’t really have a choice. You’re just admitted, so you’re either there, or you leave against medical advice. And so, this is offering patients a different avenue to receive care, but in their natural environment.

*—Nurse Manager, Leadership Staff Member, Florida*

I had a gentleman that I talked to—a Wisconsin patient. I said, “Tell me about the program. What was it like for you?” And he said, “I’m going to be honest. You are on my turf, so I’m the boss. When I’m in the hospital, you’re the boss.” This felt different to me, and I was like, “That’s what we want.” You’re giving back the patient some autonomy that, as much as we work to try to not have them lose in the hospital, they are sort of at the mercy of our scheduling departments and various testing.

*—Nurse Administrator, Leadership Staff Member, Florida*

Theme 2: Care Coordination

**Figure 2:**
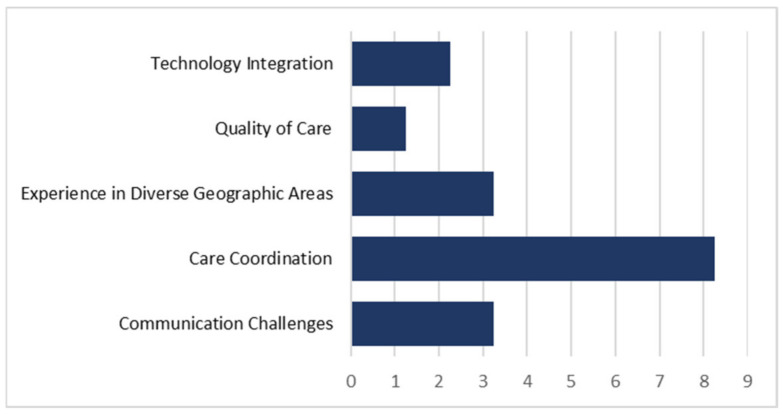
Experience frequency pertaining to the five identified themes from answers to “What challenges or barriers do you face in delivering ACH?”.

While the opportunity to look inside patient home environments offers new insights, it is not without new challenges to coordinating care. All participating staff members cited care coordination as the main challenge in delivering hybrid H@H (Figure 2).

The ACH program at Mayo Clinic relies on departments across the enterprise, including the hospital, emergency room, specialists, paramedics, and even kitchen staff. Beyond the challenge of being a multisite, multidepartmental initiative, the novel nature of the program was cited as one reason for the program’s complexity. One staff member noted that some patients place additional barriers to care by controlling access to their homes.

Patients in the hospital will put on their very best face to be able to go home. And what we find is people are very agreeable to all the policies and procedures that we put in place… We really are at the mercy of the patient’s graciousness. I know, as providers we have faced a fair number of challenges where patients will say, “No, you can’t come in my home at X time. I’m tired. Come back later.” But, that’s not how this works. This is supposed to be just like if we were in the hospital. We could have access to that sliding glass door that would take us into the patient’s room. In theory, we should have the same level of access to the patient’s home, but the patient feels very territorial to their property as they should. It’s their home. But it really is the barrier at times to providing good care.

*—Traveling Daytime Nurse, Frontline Staff Member, Florida*

Another staff member reflected that, in the early days of the program, the eligible patient population was more challenging to deliver ACH services to, as the inclusion criteria for ACH excluded healthier and younger patients due to the scope of the AHCaH waiver. Over time, however, private insurance payers reimbursed for ACH services for a wider range of eligible patients. Compared to younger patients with fewer comorbidities, older patients are, on average, less mobile and less comfortable with technology, thereby complicating the delivery of a new hybrid H@H program during the pandemic [22,23].

We, for the longest time, had very limited insurance that we could pull from initially. Let’s say I’m your 85-year-old Medicare patient with 20 different comorbidities and my spouse at home has dementia. Nobody can take care of themselves [in that home] versus a young, relatively healthy person who just happened to scratch their leg on a hike up a mountain and now has cellulitis on their leg. She can walk and talk and feed herself. She is going to be an easier patient. Let’s say she has Blue Cross Blue Shield. Well, we didn’t have access to the younger demographic for the longest time.

*—Traveling Daytime Nurse Practitioner, Frontline Staff Member, Arizona*

Coordinating care for patients from lower-SES groups has been demonstrated to be more challenging, relative to those with higher SES [24]. Yet, a paucity of information about H@H patients and demographic and socioeconomic status (SES) exists. Specific to home-based care programs in the US, which have primarily served the Medicare population, Freedman et al. (2004) found that, while Medicare patients of low SES did not have reduced access to home health services, their use of technology associated with at-home care was lower for patients who did not finish high school [21,25]. Furthermore, a recent study on patients enrolled in a Centers for Medicare and Medicaid Innovation demonstration of H@H found that H@H care may be even more beneficial for patients of low SES, such as younger Medicaid patients, due to the “ability of H@H providers to directly observe and provide care to patients in their homes, where they can address social determinants of health (e.g., food insecurity, medical equipment needs, and management of chronic diseases in real-world situations)” [26]. Here is one staff member illustrating a relevant challenge associated with ACH relative to in-person hospital care:

I try to explain to people that this is not hospital medicine. This is a hybrid. It’s not EMS. It’s not hospital. It’s a hybrid. And you’re going out into the public, you’re going out into their homes, and things don’t always work the way they do in a hospital. It’s not hospital-based medicine… I mean, it doesn’t matter what your socioeconomic background is, things can happen in anybody’s home.

*—Paramedic, Frontline Staff Member, Florida*

For hospitals to deliver care to patients across the SES spectrum, identifying new roles for ancillary team members to coordinate care in hybrid H@H is critical. Finding, training, and retaining staff who fill these gaps remain challenges to care coordination in ACH, partly due to traditional clinical practice and state policies.

We don’t integrate paramedics well into the brick and mortar [hospital]. And I will say that that is a very underutilized skill. They are wonderful members of the team. I was an ER/trauma nurse, so I worked with them both when they were coming in from the field and in the ER. But in in-hospital level of care, they haven’t been widely utilized, so [having them in ACH] has been wonderful.

*—Nurse Administrator, Leadership Staff Member, Florida*

At the other sites, paramedic visits were allowed to be substituted for nursing visits as part of the [AHCaH waiver’s] required two in-person visits every day. Here, in Arizona, the rules required that those visits be done by an RN and not a paramedic; so, that added to our vendor challenges [until that] law changed in October of 2022. So, now, paramedic visits also can count as some of those in-person visits.

*—Physician, Leadership Staff Member, Arizona*

In Arizona and Florida, three staff members noted that the paramedic shortage and outside pharmacy coordination impact the delivery of care, especially for patients who live the furthest from a clinical site or in a less-populated area and are discharged from the hospital at night.

The only hands-on [staff] we have at night are paramedics. Last week, I had a patient who was having issues, and both paramedics ended up at the house for a couple hours. Everything was delayed after that for every other patient in the program, and then they’re all dissatisfied and getting upset at us, so I think the biggest dissatisfier is for the patients that I see at night, when they’re transported so late and the transport keeps getting delayed and delayed; then, they get home and their meds aren’t there… or we can’t get their antibiotic or we didn’t get the CAD pump.

*—Command Center Overnight Nurse, Frontline Staff Member, Florida*

Beyond a shadow of a doubt, having medications delivered in a timely manner, making sure that we have access to IV medications when we need them, and making sure that our pharmacy vendor understands that these are very sick patients, and we can’t miss doses of medication. Most of my time, aside from direct patient care in the home, is spent trying to figure out why medications that I desperately needed are not in the home.

*—Traveling Daytime Nurse, Frontline Staff Member, Florida*

It’s getting better, and it’s mostly been infusion therapies that have been really hard to coordinate and get our outpatient pharmacies on board with thinking of this as hospital care, not nursing home care. They’re used to sourcing things to nursing homes and rehabs, so it’s a little bit of a different beast.

*—Traveling Daytime Nurse Practitioner, Frontline Staff Member, Arizona*

According to staff members, another layer of complexity in ACH care coordination is the transition from inpatient-level care, or acute care, to restorative phase care. After the acute episode of care is completed, patients who require rehabilitation therapy to return to their home transition to restorative care. The challenge of transitioning a patient from hospital-level care to restorative care is illustrated in one staff member’s experience:

To be in the restorative phase, you have to have met discharge criteria. When we think of the acute phase, it should model that of brick-and-mortar; so, in essence, it should be the same, despite the location. And then restorative, we think of that as they’ve been discharged. They’ve been sent to their—whatever location (we would presume home), with sometimes having home health services and sometimes not. Sometimes, we uncover that the patient should have maybe been in a rehab or a skilled nursing facility because maybe they’re not back to baseline and are not able (or their caregiver is not able) to care for themselves as much.

*—Nurse Manager, Leadership Staff Member, Florida*

Restorative care is a key component of preventing hospital readmissions [9]. By helping patients to rebuild their strength and agency, restorative care functions as a way to improve long-term patient outcomes associated with H@H. Better long-term patient outcomes with H@H play an essential role in securing payer and policymaker support for H@H programs. One staff member stressed that obtaining the support of policymakers is a high-priority goal for the H@H community to grow in different parts of the United States. Beyond securing policymaker support for ACH, the need for clarity regarding reimbursement for the transition phase to restorative care is urgently needed. When a patient transitions from hospital-level care at home to restorative care, the reimbursement process for care becomes murky. This affects the coordination of at-home restorative care services that vary by patient location, insurance, and other factors.

[Patients are] worried about how this is going to be paid. And we don’t really know, and we’re not sure if anyone really knows. You could say, “Call your insurance company,” but they’re not going to know probably what ACH is and how they are going to get billed for it because it’s brand new… I know as a frontlines nurse, sometimes, the one in that living room, in the kitchen, face-to-face with the patient, trying to talk them through all this when we sometimes or very rarely have any of the answers. I wrestle with that a lot. How do we provide that confident and professional level of care while still not overpromising because you don’t want to overpromise.

*—Traveling Nurse, Frontline Staff Member, Wisconsin*

In contrast to providers who deliver in-person care, command center staff members face unique challenges with learning new operations skills to coordinate care delivery across cities using differently skilled team members and dynamic resources.

If it’s, say, a lab collection, like for a [central venous catheter], you can either have a nurse do it, you can have a paramedic do it, or you can have a phlebotomist do it, depending on when it’s needed in the day for the patient. The service coordinator can make the judgment call to say, “OK, we’re just going to have phlebotomy go out to do this collection,” but if they see the paramedic is available, and they’re also going to the patient’s home, they can bundle that activity while the paramedic’s in the patient’s home doing the assessment, maybe administering medication, they can also collect that blood. Then, either they could deliver it back to the Mayo Lab or we would have a courier service go pick up the lab [draw] from the patient’s home and bring it back.

*—Nurse Coordinator, Frontline Staff Member, Florida*

Theme 3: Experiences in Diverse Geographic Areas

Expanding on the dynamic nature of patient home environments and SES, nine staff members voiced their opinion that rural and urban ACH delivery are different endeavors. Multiple rural staff members contrasted personal experiences being part of a community practice compared to those prevalent at a destination medical center (MCF).

I think there’s a big difference between a destination medical practice and a rural community health system. I think probably the best example is one of our very first patients had a really challenging socioeconomic situation. Her home was not the ideal home situation, as many, many of our patients experience. She had a lot of cats. There was animal excrement all over the home. I think there were some strange relationships in the family. And again, this is my perception—I think our team’s perception was this is really unfortunate and also common, and what can we do to help her be successful here? I think in Wisconsin, we are reporting less than ideal things—cigarette smoke, urine on the floor, a lot of cats—but how do we problem solve our way through that? I think on the Florida side, it was sort of like, “Well, that’s not a safe environment, so we need to discharge her, or she needs to go to a different place for care.”

*—Nurse Manager, Leadership Staff Member, Wisconsin*

As referenced in the story above, the geographic location of patients in ACH can modulate their outcomes. Ma et al. (2022) found that rural home health agencies, which were less likely to be for-profit and more likely to provide services to both Medicare and Medicaid beneficiaries, outperformed urban agencies on “timely initiation of care over time”, as well as reduced hospitalization and emergency visits over time [27]. This need to combine situational awareness of patient daily life with clinical decision making to improve patient outcomes, which is common in rural home health care, was vocalized by two rural staff members:

Our patients are not the same patients that are in Florida, and they do not have the same problems as the patients in Florida. Some of our patients can’t afford their medicines. It seems they don’t have that issue in Florida, and they have a hard time dealing with that, or it takes them a week to figure out the patient didn’t get that med, not because they were trying to cause problems, but because they couldn’t afford that inhaler that week.

*—Traveling Nurse, Frontline Staff Member, Wisconsin*

I’ve never been to Jacksonville, so I don’t know. I’m sure they have poverty because there’s poverty everywhere, but it might look different. Northwest Wisconsin rural poverty definitely is a thing, and we both came from critical access hospitals, and we both worked in the ER. We saw a lot of just really sad, desperate situations. And there is no social support, like even just trying to get somebody a cab out of the ER who’s drunk in the middle of the night. I know you go to a big city, you can get a cab or an Uber, a Lyft, 24/7, without any effort at all, generally. And up here? Yeah, good luck trying to get a cab past 9 p.m., much less any other sort of thing, like non-medical transport. In a big city like that, they have vascular access services that can do it in the home, whereas, up here, as far as I know, I’m the only nurse in the area that’s ever done vascular access in somebody’s living room.

*—Traveling Nurse, Frontline Staff Member, Wisconsin*

Urban areas are not without challenges, mainly because the coordination of care is more complex for those patients who live farthest from a Mayo Clinic campus. The timely delivery of their ACH services is modulated by vendor schedules, staffing options, and traffic.

Where the challenge can occur is that if you have one patient on one side of town and another patient on the other side of town, and they both need infusions within an hour or two of each other. The infusion lasts for about an hour. You are most likely going to be late, and so you have to give the patient a heads up. “OK, I know it’s scheduled on your appointment here, that they’re going to be there at 9. They’re most likely going to be there closer to 10 or after 10.” You have to be able to communicate that. It’s like when you’re waiting for a cable provider to come to your home.

*—Nurse Coordinator, Frontline Staff Member, Florida*

Regardless of rural or urban site, changing state and federal policies surrounding the delivery of a multistate hybrid H@H influenced staff experiences and led to increased confusion. Two staff members noted that changes regarding which providers can deliver ACH services vary by site. One staff member added that Arizona, Florida, and Wisconsin had different scope of practice regulations for paramedics. Wisconsin and Florida also have unique telehealth regulatory and policy landscapes, and, as temporary flexibilities expired in Florida during 2021, staffing roles in ACH were reconfigured [28]. One staff member also noted that the volume of regulations associated with home healthcare was extremely burdensome. Another noted that outpatient pharmacy and home health services had to be contracted with outside vendors in Arizona and Florida. Staff at all sites felt the strain of irregular state and institutional policy landscapes.

The rate of change is so, so fast that I can’t keep up, and I live it every day… these workflows change daily sometimes… so you show up to work, and 75 things have changed since your last shift. The fatigue that comes with trying to—it’s fine to do that for a period of time, but it’s been a year-plus of it… We haven’t had a month where there hasn’t been a significant change that has impacted multiple workflows, our frontline staff, everything that we do. And this has been going on for 13-plus months.

*—Nurse Administrator, Leadership Staff Member, Wisconsin*

The state of Arizona developed a [H@H] pilot program through the Arizona Department of Health Services, and it has rules tied to it. The actual rules for the state are 30 min from the base hospital. That’s obviously a very vague term. Is that 30 min in rush hour? Is that 30 min in the middle of the night?

*—Physician, Leadership Staff Member, Arizona*

Despite evidence in support of hybrid H@H programs, their implementation in a new or different geographic location cannot be assumed to be immediately effective. Here, one staff member relayed her experiences with adapting to the dynamic environment of a multistate hybrid H@H program:

I think there’s a lot of uncomfortableness in ACH because it is all foreign and new, and the leaders are learning with the frontline staff. And that can also probably be hard for frontline because frontline looked to the leaders to have all the answers, and we don’t have all the answers all of the time. We make our best assumption of how we think things should be. But at the end of the day, it might not be right, and it might not meet the needs of everyone, so it’s just trying to meet the needs of the greater group, which is not easy. I guess getting more comfortable with the uncomfortable is really important.

*—Nurse Manager, Leadership Staff Member, Florida*

Theme 4: Communication Challenges

While new hybrid workflows facilitated real-time communication, they often did so without context since communicating staff members had never met in person.

The way we interact is different. It’s virtual. I would say 95 percent of the staff, probably higher, have never met each other in real life, and that’s really hard, because there is this level of disconnect, you know, that we’re working on improving. But, when you start off virtually the way we have, it’s pretty rough.

*—Nurse Manager, Frontline Staff Member, Florida*

Two interviewed staff members noted that collaboration with lateral staff was normally easier to engage in when staff members had met in person and established rapport with one another. The all-virtual nature of some aspects of ACH team collaboration during the pandemic led to conflict. Here, another staff member expressed dissatisfaction with the hybrid workflows not allowing for direct communication between hospital staff and vendor staff being discharged to a patient’s home.

I have a middleman on everything… I can’t do my job and I can’t tell [the paramedic] exactly what I want done through an [outside] service coordinator who doesn’t have medical experience. It’s a risk I don’t want to take. I want to speak directly to the paramedic.

*—Traveling Daytime Nurse Practitioner, Frontline Staff Member, Arizona*

All ACH staff members expressed changing roles during their tenure in ACH due to different reasons; however, some staff expressed confusion about their roles in care delivery due to a lack of communication, a factor contributing to increased stress and decreased effectiveness [10,28]. One staff member shared confusion about the role of an in-person nurse versus a remote command center nurse:

I think one thing that we’re really grappling with is how do you leverage the skills of a nurse in the home to not compete with the skills of the virtual nurse, but to complement the skills of the virtual nurse? I think it’s a new model of care, and nobody’s figured it out, and it feels right now very much like we’re sort of fighting over who should do what, when probably there’s an opportunity for us to just come together and say, like, “I’m the nurse in the home. I’m the person that should do the respiratory assessment because I can actually listen to the patient’s lungs.” Like, you’re the nurse that should do this other assessment. I think it’s really important that we’re working on better defining those roles, not just for my own employees’ satisfaction.

*—Nurse Manager, Leadership Staff Member, Wisconsin*

The scaling of healthcare services in ACH to include specialty care also complicates issues around communication among teams. One staff member noted that some specialties can adapt more quickly to hybrid H@H than others, ensuring that all participating staff are on the same page, at the same time:

There are some specialty groups that have jumped on to the [ACH] model quicker than others, and then there are some logistics that still have to be ironed out, but we’ve done post-kidney transplant patients, post-bone marrow transplant patients. Those are very high acuity, complex medical conditions. There’s no way to do it without everyone working together. With our bone marrow, we have a separate rounding time so the entire oncology team can be part of the rounds, as well as our hospitalists in the command center, my team leaders, managers, and so forth. Everyone’s hearing the information at the same time, and everyone knows what the next steps are.

*—Nurse Administrator, Management/Leadership Staff Member, Florida*

### 3.3. Experiences with Using New Technology to Monitor Patients at Home

Theme 5: Technology Integration

In the ACH program, patients communicate with the command center team via telehealth videoconferences multiple times each day while patients are in the acute phase of treatment. Additionally, the use of different types of remote monitoring tools to capture and stream patient vital signs in real time has demonstrably facilitated more rapid detection of patient deterioration than at-home care without such tools [29]. Staff unanimously expressed their belief that ACH can deliver noninferior or improved patient outcomes, relative to in-hospital care, due to the integration of cutting-edge technology.

[With video telehealth,] you can take a little bit more time to get to know the patient to learn how they do things in their own home. In the hospital, we don’t see that. We discharge them, but we often don’t know what happens once they leave. Are they successful with the directions they’ve given? Can they follow the regimens that they’ve been provided? You don’t hear about it until they get readmitted.

*—Nurse Administrator, Leadership Staff Member, Florida*

Obviously, in-person care is different because it’s not—you know, you can’t hit a call light and have somebody in your room within three minutes. But I would almost—I would dare to say that their interaction with staff is probably quicker response time because they have a phone. They have an iPad. They get a lot quicker response to things than-than they would in the brick and mortar.

*—Traveling Nurse, Frontline Staff Member, Wisconsin*

[In the hospital, as a patient,] you hit your call bell and you have to wait for someone to answer. And it’s usually the hawk that’s at the front. Then, they have to get your message out to the nurse or the patient care tech. And then, you have to wait for that person to come to your room to meet your needs, whereas, for ACH, when you hit that [virtual] button, you are able to actually talk to a nurse right away. You actually get your needs met right away.

*—Nurse Coordinator, Frontline Staff Member, Florida*

With Bluetooth stethoscopes, being able to have whoever’s in the home and have the physician or provider in the command center be able to hear what they’re hearing, I think that has been really instrumental for the physicians. Bedside ultrasounds, right there, done in the home so that our team can see it at the same time, that’s a new adaptation that’s been great. Bluetooth temperature monitoring has been really helpful. We are also looking into technology that can help detect falls and, a lot of times, they don’t consider it a fall, so we’re learning more about that.

*—Nurse Administrator, Leadership Staff Member, Florida*

Although staff members highlighted technology use cases that increased the speed of healthcare decision-making, they reported general frustration with new or malfunctioning technology showing up in their workflows. Four staff members mentioned that time spent troubleshooting technology should be spent with a patient. Another staff member mentioned that multiple barriers exist to trying new technology in their program. Here is one staff member illustrating the lack of user-friendly software for placing orders:

Over time, the ordering process has improved, but that duplication is very scary to practice with because you know when you have to duplicate it can lead to errors, human errors.

*—Traveling Daytime Nurse Practitioner, Frontline Staff Member, Arizona*

Such delays associated with new technology use were expected from the get-go in ACH, especially due to its tri-site approach. Since its debut in 2020, ACH has relied on an agile development approach to software feature integration and repairs and, as of 2023, has improved many tools, as noted by multiple staff:

Apparently, our site was the first site that went to one of the new tablets. And something wasn’t working in the new tablets. For like a month or two, there was this really challenging issue where the video wouldn’t pop up for the video visits. It would pop up if you connected one way, but if you connected another way, it wouldn’t, and it was just something that you know. It didn’t get escalated very quickly. Finally, when the advanced practice providers brought it to my attention, that it still hadn’t been fixed, one or two emails get sent out and, within a day, the problem was solved. Anytime you’re on the edge of what’s the norm, you’re going to face challenges like that.

*—Physician, Leadership Staff Member, Arizona*

The software is 100% different than it was when we first went live. It was more of a portal of contact with the patient, similar to a video call and some vitals that you could save. Now, it has evolved to the nurses scheduling through that software… We meet a couple times a month, and I have frontline nurses on there, as well as my nurse manager and a nursing program coordinator, to make sure that we’re not only hearing the voice of the frontline users, but also achieving common goals for both us and our [platform vendor]. It has to work for everyone, but most changes that have occurred have been the result of the nurses’ voice.

*—Nurse Administrator, Leadership Staff Member, Florida*

## 4. Discussion

This study examined the intimate experiences of hybrid H@H staff members in three states who delivered care to patients at home during the COVID-19 Public Health Emergency. This study is the first to qualitatively explore the experiences of staff with hybrid H@H care delivery during the COVID-19 pandemic. Although novel in its focus, this study was limited by the number of participants (n = 14). One strength of this study is that its findings have significant implications for hybrid H@H programs and future research in this evolving area, including the evaluation of the AHCaH waiver by the US Department of Health and Human Services. The focus group interview format allowed staff participating in a highly collaborative program to discuss shared experiences that resulted in the discovery of themes relevant to this research area and that might not have been able to be identified via other methodologies. Staff members were able to share their individual and location-based experiences regarding communication challenges, care coordination, geographic challenges, quality of care, and technology integration that contribute to patient outcomes, staff satisfaction and retention, and long-term program sustainability.

Selection bias may have been another limitation of this study. Staff members who participated in interviews may have experienced more intense care delivery situations, making them more likely to vocalize their perspectives. Furthermore, the roles of staff who participated were likely broader than their specific titles, given that the delivery of ACH required heightened flexibility during the pandemic and with new technologies integrated into daily workflows. Thus, their experiences may not be generalizable outside of the pandemic timeline. Future qualitative studies should be conducted in non-emergency times, over longer periods of time, and with more staff members to better understand staff experiences. Moreover, more clinical trials are needed to generate evidence supporting optimal inclusion criteria for patients who would most benefit from hybrid H@H and permanent reimbursement models for hybrid H@H programs, necessary steps in advancing research for hybrid care.

Experiences with implementing a new care delivery model were linked with experiences in using new technologies to monitor patients at home by the frequent codes identified through interviews: communication challenges, care coordination, experience in diverse geographic areas, quality of care, and technology integration. Compared to previous H@H programs, those deployed during the COVID-19 pandemic were hybrid in nature, generating more data from advanced remote monitoring devices [30]. The use of these remote monitoring devices in H@H is linked to improved patient outcomes, ones that rival and, sometimes, exceed outcomes from in-hospital care [31]. Furthermore, the use of 24/7 monitoring by staff across state lines can help ameliorate staffing issues in one location, thereby helping to improve patient outcomes, as reported by participants in this study [32]. 

Regarding communication and coordination of care, while technology can facilitate better care outcomes, as in the case of enhanced remote monitoring, it can also complicate staff workflows and interpersonal communication in hybrid H@H [14]. While not specifically outlined as a technology issue in this study, the use of electronic health records (EHRs) to identify eligible patients for hybrid H@H during the pandemic was a time-consuming endeavor that required multisite and multiteam collaboration [14]. 

Overall, staff reported that the quality of care delivered was, at minimum, comparable to in-hospital care, and that patient satisfaction appeared higher in hybrid H@H compared to in-hospital care. This finding is corroborated by the results of an investigation conducted a few years prior with over 200 patients, reporting that 99% of hybrid H@H patients were satisfied or very satisfied with the program [33]. A recent systematic review found that readmission and mortality rates for H@H programs were noninferior to in-hospital care, and that H@H programs were associated with shorter hospital lengths of stay (LOS) [31]. One consideration regarding the findings of this review is that it included both traditional and hybrid H@H programs.

Detailed knowledge about a region, a community, a neighborhood, and a patient is essential to delivering high-quality, patient-centered care at home. In rural and sparsely populated areas, where community providers are more common, this intimate method of practicing medicine is the standard [34]. A recent study on health equity in hybrid H@H programs operated during the pandemic found that lower-socioeconomic-status patients may obtain even greater benefit from hybrid H@H programs than their counterparts [35]. While little is known about the intersection of patient socioeconomic status and H@H programs, the results of our study suggest that a granular understanding of the types of poverty unique to a local population receiving hybrid H@H is a necessary exercise to ensure future hybrid H@H programs do not exacerbate health disparities in the US [35]. 

## 5. Conclusions

This three-site qualitative interview study allowed researchers to learn about provider experiences in one health system delivering care in urban, rural, and desert settings. By interviewing ACH providers at three sites, this study identified themes related to H@H and hybrid care from the perspective of personnel delivering the care. As an exploratory work, this study highlighted areas for future research at the intersection of hybrid H@H and staff satisfaction. 

The findings presented in this paper could be tailored to meet the unique demographic and geographic needs of health systems located in individual states and regulated by unique home health and telehealth requirements. Understanding that hybrid H@H staff not only have shared experiences across states, but also unique site- and state-specific experiences is critical to understanding the unique challenges hybrid H@H staff face. According to the information aggregated in this paper, building a sustainable H@H program in the digital age requires hands-on knowledge of the complex situations, modulated by state and geography, in which staff find themselves. 

As hybrid H@H programs scale to provide specialty care, the complexity of care coordination increases. While Mayo Clinic is a health system with the resources and training to facilitate cross-site collaborations, not every health system will be poised to support as wide a range of care services via hybrid H@H. Regarding the transition of patient health status from acute to restorative, or from acute to specialty care, the need for clarity on reimbursement and regulatory issues is integral to ensuring that hybrid care improves long-term outcomes and is a clinically useful part of care coordination.

The small sample size of staff member interviews was a limitation of this study. Moving forward, we recommend that interviews last longer than the 45 min average time period employed by this protocol to encourage open identification of specific issues by cross-site teams. We hope this study will encourage other hybrid H@H programs to conduct qualitative interviews with their staff to identify themes and challenges relevant not only to all H@H programs but also to the unique states and communities their staff serve. Lastly, this paper was an early, exploratory work for consideration by randomized controlled trial and observational research endeavors in hybrid H@H programs with the goal of enhancing the robustness of future hybrid care research.

## Figures and Tables

**Table 1:** Selected sociodemographic characteristics for focus group interviews.

Selected Sociodemographic Characteristics for Focus Group Interviews						
	Interview 1	Interview 2	Interview 3	Interview 4	Interview 5	Interview 6
Number of Participants	3	2	3	2	2	2
Sex						
Male	1	1	0	1	0	0
Female	2	1	3	1	2	2
Race/Ethnicity						
White	3	2	3	0	2	2
Hispanic	0	0	0	1	0	0
Asian	0	0	0	1	0	0
Location						
Wisconsin	3	1	1	0	0	0
Florida	0	1	2	2	2	0
Arizona	0	0	0	0	0	2
Staff Role						
Frontline Staff	2	1	0	1	2	2
Leadership	1	1	3	1	0	0

## Data Availability

The data that support the findings of this study are available from the corresponding author upon reasonable request. The data are not publicly available due to privacy restrictions.

## Appendix A. Focus Group Interview Question Prompts

(1) How did you get involved with Advanced Care at Home?
(2) What challenges or barriers do you face in delivering Advanced Care at Home?
(3) How frequently do you interact with patients in Advanced Care at Home?
(4) How frequently do you interact with other staff in Advanced Care at Home?
(5) What recommendations do you have to improve Advanced Care at Home?

## Appendix B. Codebook

**Code**	**Description**	**Quote Example**
Communication challenges	Barriers to communication within staff teams, between staff and patients, or across hospital sites/states	I think that normally [ACH] would’ve been largely virtual, no matter what the external geopolitical forces, because of the nature of the logistical structure with a command center and all these different moving pieces. It’s constant communication electronically by phone and email, and disconnected entities have cultural practice differences.
Care coordination	Participant descriptions of the complexity of coordinating care for hybrid home hospital delivery	Hospitals, as much as we don’t realize it, run pretty seamlessly. A doctor places an order for an intervention or a test, and it sort of just happens. It goes through a queue and the computer, and then you can schedule accordingly, but it’s all done behind the scenes, so nurses don’t typically see that side of things. We don’t really have that in the home hospital. There’s another person in the mix scheduling the appointments. The nurses in the command center found that they were in a lot of that coordination, clinically triaging what’s more appropriate to happen first and so forth: “OK, it’s really important that they get this chest X-ray, but it’s also important that they see physical therapy today.” It’s not just the click of a button.
Experiences in diverse geographic areas	Any comments related to how staff in rural, urban, or desert areas deliver care or interact with patients and other staff	I think maybe it’s easier in a destination medical practice to say, “Wait, this patient has a lot of stuff going on. They’re not a patient that we’re going to take care of,” or maybe you just don’t see those patients as much in a destination medical practice. That has definitely been challenging, as I think it’s probably hard for the Florida team to really understand the level of socioeconomic and social determinants of health impact that we’re dealing with in our patient homes.
Quality of care	Staff discussion of how the hybrid home hospital model impacts the quality of care delivered (patient outcomes and satisfaction)	We’ve been able to take a lot of people home and have them do well in their home environment and be really appreciative of the care that we get to provide in the home. I think that’s a success, providing high-level care in their house and having them be so appreciative to be home.
Technology integration	Any comments related to the use of technology in hybrid home hospital delivery	Technology, technology, and technology. Sometimes it works well. Sometimes it doesn’t work at all.
